# Dr. Knopf’s Prize Essay

**Published:** 1907-07

**Authors:** 


Dr. Knopf's Prize Essay.—I have received a pamphlet copy of this able and famous essay. It is entitled "Tuberculosis as a Disease of the Masses, and How to Combat It." It has a supplement in "School Hygiene, Home Hygiene, Installation of the Sanatorium Treatment at Home, and a Historical Review of the Anti-Tuberculosis Movement in the United States"; that is, up to the date of the meeting in Berlin, May 24, 1899, at which meeting Dr. Knopf's paper was read and awarded the international prize, through a select committee, July 31, 1900. For, a great deal has been done since that date, of which no mention is made, although on the title page it is announced that the work has been revised with supplement, 1907; e. g., no mention whatever is made of the American International Congress on Tuberculosis held in New York City, November 14, 15, 16, 1906, under the auspices of, and with the patronage of, the United States government, whose Secretary of State officially invited delegates from all foreign countries with whom we have relations. In response to this invitation, delegates were present from many foreign and most of the States of America; and notable papers were read from distinguished scientists who regretted they could not be present. Just why this omission occurs, I am at a loss to understand. The American International Congress was inaugurated in 1900, and held its first meeting in New York in June, 1900, as the American Congress on Tuberculosis, under the presidency of the most distinguished sanitarian in America, Dr. A. N. Bell, of Brooklyn. Its name was changed at the historical fourth meeting at St. Louis, October, 1904. It really inaugurated the crusade against consumption, and put in motion the forces which now are world-wide, enlisting the co-operation of sanitarians, engineers, economists, educators and the five million members of the Fraternities, etc., etc.

Aside from this seemingly invidious discrimination, Dr. Knopf has fully covered the whole field; has compiled from best works all that can be said on every phase of the subject. He brings it fully up to the present and sets forth really all that is at present known of the subject.

The essay is published by Fred P. Flori, 514 East Eighty-second Street, New York, and is for sale, also, by "Charities and the Commons," 105 East Twenty-second Street, New York. Price, 50 cents per copy, cloth; 25 cents per copy, paper cover.

